# Vaccine protection of broilers against various doses of wild-type *Salmonella* Typhimurium and changes in gut microbiota

**DOI:** 10.1080/01652176.2024.2440428

**Published:** 2024-12-25

**Authors:** Samiullah Khan, Andrea R. McWhorter, Nicky-Lee Willson, Daniel M. Andrews, Gregory J. Underwood, Robert J. Moore, Thi Thu Hao Van, Kapil K. Chousalkar

**Affiliations:** a School of Animal and Veterinary Sciences, The University of Adelaide, Roseworthy, Australia; b Bioproperties Pty Ltd, Ringwood, Australia; c School of Science, RMIT University, Bundoora, Australia

**Keywords:** Vaxsafe® ST, broiler chickens, poultry production, salmonellosis, gut microbiota, microbial diversity, dose of infection, vaccine diluent, *Salmonella* Typhimurium

## Abstract

This study evaluated the impact of vaccine diluents (peptone or water) on the protective effects of *Salmonella* Typhimurium (*S*. Typhimurium) vaccine. Vaccinated broilers were challenged with different doses of wild-type *S*. Typhimurium through dust. At the time of cull, vaccine load was highest in caeca and lowest in spleen. Wild-type *S*. Typhimurium was detectable after 24 hrs only in the vaccinated birds challenged with 108 CFU and positive control. *S*. Typhimurium load was lower in the organs of the groups challenged with 104 and 106 compared to the 108 CFU group. The caecal microbiota alpha diversity of the vaccinated or vaccinated and challenged chickens differed from the positive and negative control groups. Beta diversity of the positive control clustered separately from all other treatment groups, showing that vaccine caused minimal changes in gut microbiota structure. The vaccinated and/or wild-type challenged chickens showed significantly higher abundance of *Anaerostignum*, *Lachnoclostridium*, *Intestinimonas*, *Colidextribacter*, *Monoglobus*, *Acetanaerobacterium* and *Subdoligranulum*. Outcomes from this study demonstrate that the vaccine effectively protected broiler chickens from *S*. Typhimurium infection and helped maintain a more stable gut microbiota structure, reducing the impact of *S*. Typhimurium on gut health. Vaccine diluent did not affect gut microbiota composition.

## Introduction

Globally, the consumption of chicken meat is increasing. One reason for this increase is the lower retail cost of chicken compared with lamb, beef, pork or fish (Adamski et al. 2017; Milford et al. 2019). In 2023, the global broiler meat production was around 103 million metric tons. According to the Australian Agricultural Commodity Statistics, for the year 2021/22, 701.4 million broiler chickens were slaughtered to produce 1350.4 thousand tons of meat. In Australia, the per capita chicken meat consumption for the year 2022/23 was 50.1 kg.

Poultry can become persistently infected with *Salmonella* serovars pathogenic for humans and thus pose a threat to public health. Trace-back investigation of Australian human salmonellosis cases has shown that contaminated poultry meat plays an important role in the transmission of human pathogenic *Salmonella* serovars, such as *Salmonella* Agona, *Salmonella* Typhimurium, *Salmonella* Virchow and *Salmonella* Infantis (Ford et al. 2018; McLure et al. 2022; Padrotta et al. 2022). To reduce the overall microbial contamination of chicken meat, in Australia and some other regions, broiler carcasses are washed in chlorine treated water in the processing plants (Chousalkar et al. 2019). However, the load of *Salmonella* on chicken carcasses and the accumulation of organic matter in the chill tanks can contribute to *Salmonella* persistence on meat and chicken meat products, representing a potential health risk to humans who consume the products. *Salmonella* can also survive on processing plant equipment (Obe et al. 2020), which may lead to the cross contamination of subsequent batches. Therefore, on-farm strategies are important to mitigate *Salmonella* levels in the chicken meat supply chain.

On-farm intervention strategies include poultry farm and hatchery decontamination (McMullin 2009; Oastler et al. 2022), biosecurity protocols (Totton et al. 2012), use of feed additives (Abudabos et al. 2019; El-Saadony et al. 2022; Hu et al. 2023) and vaccination (Han et al. 2020; Acevedo-Villanueva et al. 2022). Most of the chicken meat production in Australia is vertically integrated, where companies own breeding flocks, hatcheries and broiler farms (Crabb et al. 2018). In broiler production, the all-in all-out strategy is a common practice with a grow out period of approximately six weeks. Post-production, farm sheds are decontaminated by producers through a subcontract process. In addition to decontamination, and biosecurity protocols, vaccination is often recommended to reduce *Salmonella* loads in birds as well as the farm environment. *Salmonella* vaccination in broilers is not practiced in Australia due to their short life span. However, vaccination in broilers has been shown to reduce the colonisation and shedding level of *Salmonella* (Han et al. 2020; Acevedo-Villanueva et al. 2022; Cookson et al. 2024). Vaxsafe® ST, which is a live- attenuated *Salmonella* Typhimurium vaccine, is recommended for vaccinating broilers in Australia. Vaxsafe® ST injection in broilers significantly increased the immune response measured at 14 days post-vaccination (Groves et al. 2015). Although, in Australia, broilers are not widely vaccinated against *Salmonella*, vaccination in broilers has been identified as an important preharvest intervention for *S*. Typhimurium control on chicken meat carcasses. This study was conducted to test vaccine efficacy in broilers when chickens were introduced to varying levels of wild-type *S.* Typhimurium.

Within the horizontal route of transmission, airborne infection of chickens with *Salmonella* has been established (Holt et al. 1998). Dust plays an important role in the transmission of *Salmonella* in a chicken shed (Pal et al. 2021). Poultry shed dust is comprised of particles from litter, faeces, feed, feathers etc. Dust from the layer poultry environment has been shown to contain variable loads of *Salmonella* (Gole, Caraguel, et al. 2014; Gole, Torok, et al. 2014; Sharma et al. 2018). In broiler production, dust has been shown as a source of *Salmonella* (Pal et al. 2022). *Salmonella* can transfer from contaminated litter to dust in a shed environment. For example, an inoculated litter with increased levels of *Salmonella* Typhimurium resulted in an increased probability of positive dust samples (Pal et al. 2021). Reduction of dust in poultry shed results in a decrease in bacteria (Mitchell et al. 2004). In a poultry shed, dust settling on surfaces such as windowsills, walls and fan blades can be a source of *Salmonella*.

The gut microbiota influences the overall performance and health of broiler chickens (Stanley et al. 2014). Ideally, a vaccine should not induce changes in the composition of gut microbiota that negatively impact host health. There has been limited investigation of the effects of Vaxsafe® ST on poultry gut health. Thus, it is largely unknown how vaccination with a live attenuated *Salmonella* strain affects the gut microbiota of broilers. In a recent study, three different types of *Salmonella* vaccines were administered to broilers to investigate the differences in their abilities to produce changes in the composition of gut microbiota (Lyimu et al. 2023). Among them, the vaccines AviPro *Salmonella* DUO and AviPro *Salmonella* Vac T but not AviPro *Salmonella* Vac E significantly changed the composition of gut microbiota (Lyimu et al. 2023). Two differently modified strains of *S.* Typhimurium (P_BAD_-mviN and DDmetRmetD strains) affected the composition of caecal microbiota differently (Park et al. 2017). Therefore, investigating the effects of Vaxsafe® ST on gut microbiota composition in the presence or absence of wild-type *Salmonella* challenge in broilers will enhance our knowledge about the usefulness of this vaccine for control of *S.* Typhimurium in meat type chickens.

Vaccine efficacy is affected by multiple factors, such as route of administration, vaccine candidate, gut colonisation ability, and vaccine diluent. Based on current recommendations, Vaxsafe® ST is re-constituted in water and can be administered to day-old chicks. Vaxsafe® ST has been generated from the parent strain by mutation of *aroA* gene by transposon mutagenesis (Bioproperties n.d.). This mutation renders Vaxsafe® ST unable to synthesize aromatic amino acids as a part of normal cell metabolism (Alderton et al. 1991; Bioproperties n.d.). To keep the metabolic activities of Vaxsafe® ST optimum, these amino acids can be partly supplied by reconstituting the vaccine in a nutritive media. Thus, we hypothesized that a nutritive vaccine diluent might prime the vaccine for better colonisation required for stimulating the mucosal immune system. Therefore, we aimed to test a plant-based peptone as diluent on vaccine efficacy in broilers. The main objective of the current study was to evaluate the extent of vaccine protection in broilers against different doses of wild-type *S.* Typhimurium when the vaccine was reconstituted in different diluents. Gut microbiota was investigated by 16S rRNA gene amplicon analysis to understand variation in the vaccinated and wild-type *S.* Typhimurium broiler chickens.

## Methods

### Animal ethics statement

The Animal Ethics Committee at the University of Adelaide approved the broiler chicken experimental work (approval number S-2020-076) in accordance with the guidelines specified in 'Australian code for the care and use of animals for scientific purposes, 8th edition (2013)'. The study followed ARRIVE guidelines required for *in vivo* experiments (Percie Du Sert et al. 2020).

### Hatching and rearing of broiler chicks

Broiler chickens (Cobb 500) of mixed sex (*n* = 90; nine/treatment group across two pens) were hatched and reared at the School of Animal and Veterinary Sciences, Roseworthy Campus as per the standard guidelines in the Cobb Broiler Management Guide. Meconium samples from hatching trays of the incubator were tested for *Salmonella* spp. following the method described previously (Khan and Chousalkar 2020) to confirm the *Salmonella* free status of the hatched chicks.

### Vaccine reconstitution in appropriate diluent and chick vaccination

A single vial of live attenuated *S.* Typhimurium vaccine (Vaxsafe® ST) was either reconstituted in 1 mL broth of peptone from soymeal (with 2% glucose at final concentration) or sterile water diluents pre-warmed at 41 °C. Each vial contained a dose of 10^10^ colony forming unit (CFU) per mL. The peptone broth (same batch; CAS-No: 91079-46-8) was prepared as per manufacturer's instructions (Sigma). Peptone from soymeal was used as a diluent, as there were no quarantine/biosecurity restrictions on use due to its originating source. The reconstituted vaccine vials were diluted to 10^8^ CFU/mL, incubated at 41 °C for 30 min, and a 100 µL was orally inoculated into individual chicks (dose of 10^7^ CFU/bird). Unvaccinated control groups received 100 µL of either sterile peptone or water (Table 1). The chicks were vaccinated at day 1 of hatch and reared in pens. The pens contained chick paper and wood shavings. Throughout the trial the chickens were fed Meatline Starter crumble that contained lasalocid sodium as coccidiostat. Cloacal swabs were collected at days 3, 7, 14, 17, and 20 post-vaccination to characterise vaccine shedding using bacteriological culture enrichment method. In the same samples, bacterial load was determined using qPCR. Cloacal swabs collected at days 14, 17, and 20 were also processed for wild-type *S.* Typhimurium using culture methods and qPCR (Table 1).

**Table 1.** Treatment group details of broiler chickens for ­Vaxsafe® ST vaccination and wild-type *S.* Typhimurium challenge.

Treatment	Vaccination status and diluent	Wild-type *S.* Typhimurium challenge dose
Negative control	Unvaccinated	Unchallenged
Positive control	Unvaccinated	10^8^ CFU/pen
Vaccinated control	Vaccine reconstituted in peptone	Vaccinated control peptone diluent
Vaccinated and 10^4^ CFU ST challenge	Vaccine reconstituted in peptone	10^4^ CFU/pen
Vaccinated and 10^6^ CFU ST challenge	Vaccine reconstituted in peptone	10^6^ CFU/pen
Vaccinated and 10^8^ CFU ST challenge	Vaccine reconstituted in peptone	10^8^ CFU/pen
Vaccinated control	Vaccine reconstituted in water	Vaccinated control water diluent
Vaccinated and 10^4^ CFU ST challenge	Vaccine reconstituted in water	10^4^ CFU/pen
Vaccinated and 10^6^ CFU ST challenge	Vaccine reconstituted in water	10^6^ CFU/pen
Vaccinated and 10^8^ CFU ST challenge	Vaccine reconstituted in water	10^8^ CFU/pen

Each chick received the respective vaccine dose orally in a 100 µL volume, while vaccine diluent control groups received 100 µL of sterile diluent (water or peptone). Chicks were vaccinated at day 1 of hatch and selected groups were challenged with wild-type *S.* Typhimurium at day 13 post-vaccination. In each treatment group, there were 9 chickens. All the chickens were culled at day 20 post-vaccination.

### Broiler challenge with wild-type S. **Typhimurium** inoculum prepared in dust

For the *S.* Typhimurium challenge,10^4^,10^6^, and 10^8^ colony forming units (CFUs) of *S.* Typhimurium per gram of dust were prepared and sprinkled in the appropriate pen containing 4 or 5 chickens at day 13 post-vaccination. Briefly, a stock culture of wild-type *S.* Typhimurium isolated from broiler chickens (MLVA No: 04120000463) was revived on nutrient agar and a single colony was sub-cultured in Luria Bertani broth. This wild-type strain has not been studied for its invasive characteristics previously. The *S.* Typhimurium broth culture CFU was estimated from its spectrophotometric optical density reading at 600 nm and a volume containing 10^8^ CFU was centrifuged at 5000 ×*g* for 5 min. The supernatant was discarded, and the bacterial pellet was re-suspended in 150 µL physiological saline. The 10^6^ and 10^4^ CFU/150 µL dilutions were prepared from the 10^8^ CFU/mL culture samples. Fine particle dust collected from a broiler shed tunnel fans was autoclaved and dried overnight in an oven. It was determined that approximately 150 µL of water was sufficient to keep the water activity of the autoclaved dust around 0.8, while preserving the dust nature without making it a paste. Water activity of the dust was measured using a PawKit Water Activity Meter (Model P06760; AquaLab, Decagon Devices, United States) on a scale of 0 to 1, which represent minimum to maximum water activity, respectively. The 150 µL inoculum was added into 1 g of dust and mixed with the tip of culturing loop in a 25 mL sterile plastic container. The final inoculums prepared were: 10^4^, 10^6^, and 10^8^ CFU/g of dust (Table 1). In each pen (0.687 m^2^) of the respective treatment groups, 1 g of dust was sprinkled on a newly placed chick paper. The control groups received 1 g of dust without *S.* Typhimurium.

### Measurement of humoral immune response

At day old, prior to vaccination, blood was collected from 7 chicks to determine the level of maternal antibodies. At day 19 post-vaccination, 6 blood samples from each treatment group (Table 1) were collected. All the blood samples were processed for IgY antibody quantification using *Salmonella* Group B ELISA kit (BioChek). The ELISA was performed as per the manufacturer's instruction on 1:500 diluted plasma samples.

### Vaccine and wild-type S. **Typhimurium** load determination in organs

At day 20 post-vaccination, all the chickens were humanely euthanised via the cervical dislocation method. Spleen, liver, lung, duodenum, jejunum, ileum, and caecum were collected. For consistency, samples were collected from the same area of the organ and the gut samples were taken from the middle part of each segment. Small pieces (approx. 0.1 g) of the tissue samples were collected in 1.5 mL tubes containing 0.5 mL of 0.9% saline and approx. 0.4 g of stainless steel beads (0.5–2.0 mm diameter). Samples were maintained on ice, homogenised, and serially diluted in saline. One hundred microlitres were spread plated into xylose lysine deoxycholate (XLD) agar. The agar plates were incubated overnight at 37 °C and examined for enumeration of typical colonies of vaccine and wild-type *S.* Typhimurium. Wild-type *S.* Typhimurium produces blackish colonies with mucoid halo, while the vaccine strain produces transparent glossy colonies on XLD agar media plates. Samples that were negative on direct plating were enriched using buffered peptone water (BPW) and Rappaport-Vassiliadis (RVS) broth media.

### Total DNA extraction from caecal contents and faeces

Total microbial DNA was extracted from caecal content samples (*n* = 90, 9/treatment) using a modified protocol of the QIAamp FAST DNA Stool Mini Kit (Qiagen). DNA was also extracted from faecal material collected by cloacal swabbing of individual chickens (*n* = 90) at days 3, 7, 14, 17, and 20 post-vaccination. Briefly, approximately 200 mg of caecal content or faeces per sample was weighed into a 1.5 mL safe-lock tube, and into each sample, 700 µL of Buffer InhibitEx was added. For maximum bacterial cell lysis, an approx. 0.15 g mixture of glass beads (acid-washed ≤106 μm and 425–600 μm; Sigma Aldrich) was added to the samples and homogenised in a bullet blender (Next Advances, USA) for 5 min at speed 10. The samples were processed for DNA extraction as previously described (Khan and Chousalkar 2021). 16S rRNA gene amplicons were produced from the caecal DNA samples. The faecal DNA was used for vaccine and wild-type *S.* Typhimurium quantification by qPCR.

### 16S rRNA gene amplicon sequencing and data analysis

The V3-V4 region of the 16S rRNA gene was amplified (338 F − 806 R (V3 - V4) using the forward (5′-ACTCCTACGGGAGGCAGCAG-3′) and reverse (**5′**-GGACTACHVGGGTWTCTAAT-**3′**) primer pair) with Q5 high fidelity DNA polymerase (New England Biolabs), using dual indexing and variable spacer primers. The cycling conditions for PCR were 98 °C for 1 min, 35 cycles of 98 °C for 10 s, 49 °C for 30 s, and 72 °C for 30 s and final extension at 72 °C for 10 min. The amplicon sequencing was performed using an Illumina MiSeq system (2 × 300 bp). The data was demultiplexed with the onboard Illumina software and the microbiota analysis was performed in Quantitative Insights into Microbial Ecology 2 (QIIME2) (Bolyen et al. 2019). Quality filtering, denoising, and chimera removal were performed using DADA2 (Callahan et al. 2016) as a QIIME2 plugin with all recommended parameters and an amplicon sequence variant (ASV) table was generated. Taxonomy was assigned using SILVA v138.1 database (Quast et al. 2013). The ASV table was uploaded into MicrobiomeAnalyst for data normalization using the cumulative sum scaling (CSS) option, and the data were analysed using alpha diversity, beta diversity and ANOVA options as per recommendation of the online software (Chong et al. 2020).

### Vaccine and wild-type S. **Typhimurium** load determination in caecal contents and faeces by qPCR

*S.* Typhimurium qPCR was performed to quantify wild-type and vaccine strain DNA using the primers listed in Table 2. The primer pairs were tested for specificity and amplification efficiency using 10-fold serially diluted DNA of each strain. The qPCR was performed using SensiFAST SYBR Hi-ROX Kit (Bioline) in a 20 µL final reaction volume. The reactions contained 10 µL of SensiFAST buffer, 1 µL of each of the forward and reverse primers (10 µM), 2 µL of specific DNA template and 6 µL of water. The cycling conditions in the QuantStudio 6 Flex instrument were: initial denature at 95 °C for 3 min, 40 cycles of annealing at 60 °C for 30 sec and extension at 72 °C for 30 sec, a hold stage at 72 °C for 5 min and melt from 60 °C to 95 °C. The specificity of the primer pair was confirmed by the single peak during melt curve analysis and running the amplicon on 2% agarose gel. The amplification efficiency (AE, %) was calculated through E = −1 + 10^(−1/slope)^.

**Table 2.** Vaxsafe® ST and wild-type *Salmonella* Typhimurium specific qPCR primers.

Gene target	*Salmonella* strain	Primer sequence (5′ - 3′)	Fragment length (bp)	AE (%)	Reference
*aroA*	wild-type	F: 5′-TCTTTTTTCATCCCCACG-3′ R: 5′-CGGTTTTACCACAAGCTAA-3′	182	97	(McWhorter and Chousalkar 2018)
*aroA*	vaccine	F: 5′-GGTGTAATTGATCCCCAACG-3′ R: 5′-GGTGTAATTGATCCCCAACG-3′	204	98	This study

### Vaccine and wild-type S. **Typhimurium** DNA fragment cloning and generation of standard curve

Freshly generated qPCR products (204 bp amplicon length for Vaxsafe® ST and 182 bp for wild-type *S.* Typhimurium) were cloned (pCR4-TOPO) that were inserted into DH5α-T1^R^ chemically competent *Escherichia coli* cells as per the manufacturer's protocol of One Shot Chemical Transformation, TOPO TA Cloning Kit for Sequencing (Invitrogen). The recombinant plasmids were extracted using PureLink Quick Plasmid Miniprep Kit as per the manufacturer's protocol (Invitrogen. The insertion of the Vaxsafe® ST and wild-type *S.* Typhimurium fragments into the plasmids were confirmed by qPCR, melt curve analysis and running the amplicons on 2% agarose gel. Vaxsafe® ST and wild-type *S.* Typhimurium DNA were used as positive controls. The recombinant plasmids were serially diluted to construct standard curves for the quantification of vaccine and wild-type *S.* Typhimurium load from caecal contents and faeces. The DNA copy number for the recombinant plasmid was calculated from the plasmid DNA concentration and the molecular weight of the plasmid with Vaxsafe® ST or wild-type *S*. Typhimurium fragment inserts using the below formula.

$$\text{Number}\;\text{of}\;\text{copies}\;=\;\frac{\text{DNA}\;\text{amount}\;\left(\text{ng}\right)\;\times \;6.022\;\times \;10^{23}}{\text{DNA}\;\text{length}\;\left(\text{bp}\right)\;\times \;1\;\times \;10^{9\;}\;\times \;660}$$

Where DNA refers to recombinant plasmid DNA, 6.022 × 10^23^ is the Avogadro's constant, 1 × 10^9^ is a conversion factor and 660 refers to the average mass of 1 bp dsDNA.

### Vaccine and wild-type S. **Typhimurium** load in caecal content and faeces

Using the standard-curve based qPCR on extracted DNA, vaccine load was quantified from the caecal content collected at day 20 and faeces collected at days 3, 7, 14, 17, and 20 post-vaccination. Using the standard-curve based qPCR on extracted DNA, wild-type *Salmonella* load was quantified from faeces collected at days 14, 17, and 20 post-vaccination. qPCR was performed (in duplicate in 20 µL final reaction volume) in 384 well plate using an epMotion 5075 robot and QuantStudio 6 Flex qPCR machine. Cycling conditions for the qPCRs were the same as described for primer optimisation. Based on both the criteria for single peak of the melt curve and Cq values, samples falling out of the range (Cq > 32) were excluded for the vaccine and wild-type *S.* Typhimurium copy number determination.

## Statistical analysis

The vaccine and wild-type *S.* Typhimurium proportion of positive samples-qualitative data, CFU and log_10_ DNA copy number in caecal content and faeces data were analysed in GraphPad Prism using ANOVA and non-parametric analysis. Level of significance was determined by Fisher's LSD at *p* < 0.05.

## Results

### Shedding of vaccine and wild-type S. **Typhimurium** strains

**Figure 1.**
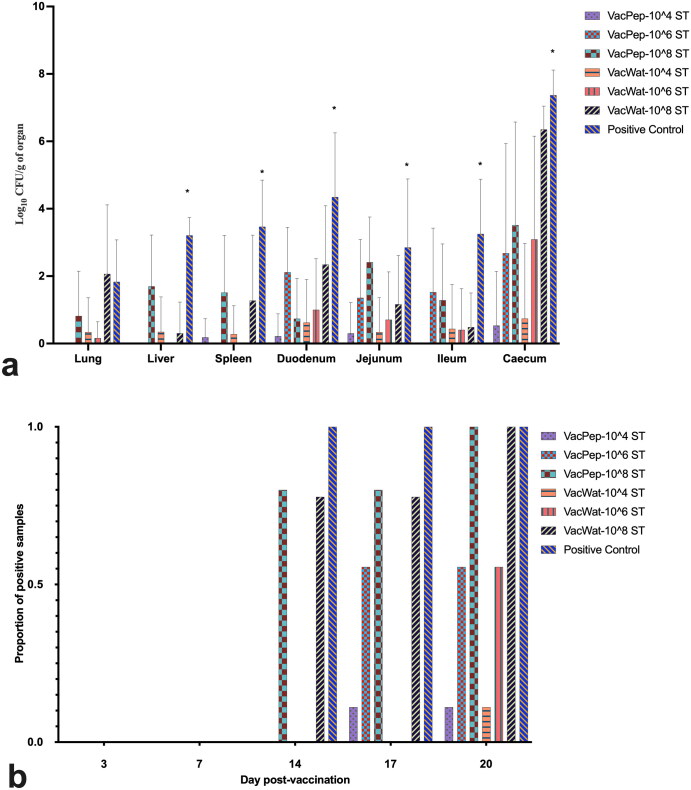
Wild-type *S.* Typhimurium detection in organs and cloacal swabs. a) Wild-type *Salmonella* load in organs at day 20 post-vaccination (day 7 post-infection). b) Wild-type *Salmonella* proportion of positive samples at days 14, 17, and 20 post-vaccination (days 1, 4 and 7 post-infection). Broilers in the respective treatment groups were vaccinated at day 1 post-hatch. Broilers were infected with wild-type *S.* Typhimurium at day 13 post-vaccination. For wild-type *Salmonella* detection, cloacal swabs were collected at days 14, 17, and 20 post-vaccination (correspond to days 1, 4, and 7 post-infection). For wild-type *Salmonella* load quantification from organs, samples were collected at day 20 post-vaccination. No wild-type *S*. Typhimurium were quantified from negative control or vaccine control treatment groups; therefore, those groups were excluded from the graphic presentation of the data. For asterisk (*) in panel graph (a) shows that the wild-type *Salmonella* load in the positive control group was significantly higher (*p* < 0.05) from any other treatment group in the respective organ.

The results from direct plating showed that the load of wild-type *S.* Typhimurium in liver, spleen, ileum, and caecum was significantly lower in the vaccinated treatment groups that were challenged with 10^4^, 10^6^, or 10^8^ CFU compared with positive control, irrespective of the diluent (peptone vs water) used for vaccine reconstitution (Figure 1a). As shown by the load of *Salmonella* in different segments of the gut, a significantly lower load of wild-type *S.* Typhimurium was quantified from the vaccinated groups challenged with 10^4^ and 10^6^ CFU. Higher *S.* Typhimurium loads were observed in the caecum compared to lung, liver, spleen, duodenum, jejunum, and ileum. The direct plating results showed that 40% of the chickens vaccinated with the peptone reconstituted vaccine and then challenged with 10^8^ CFU wild-type *S.* Typhimurium tested negative. In contrast, 100% of the chickens in the water reconstituted vaccinated group, challenged with 10^8^ CFU wild-type *Salmonella*, as well as the positive control group, tested positive (Supplementary Figure S1). All lung samples from birds vaccinated using peptone diluent and challenged with either 10^4^ or 10^6^ CFU wild-type *S.* Typhimurium were negative (Supplementary Figure S1). Overall, the organ enrichment data showed a lower recovery percentage of wild-type *S.* Typhimurium from the vaccinated chickens challenged with 10^4^ and 10^6^ CFU wild-type *Salmonella* compared with the vaccinated and 10^8^ CFU *Salmonella* challenged and positive control treatment groups. The cloacal swabs culture data showed that 24 hr post-challenge, *S.* Typhimurium was isolated from the treatment groups vaccinated and infected with 10^8^ CFU and positive control (Figure 1b) but not from the vaccinated and challenged with 10^4^ and 10^6^ CFU treatment groups. No wild-type *S.* Typhimurium was isolated from the negative and diluent vaccinated/unchallenged control groups.

**Figure 2.**
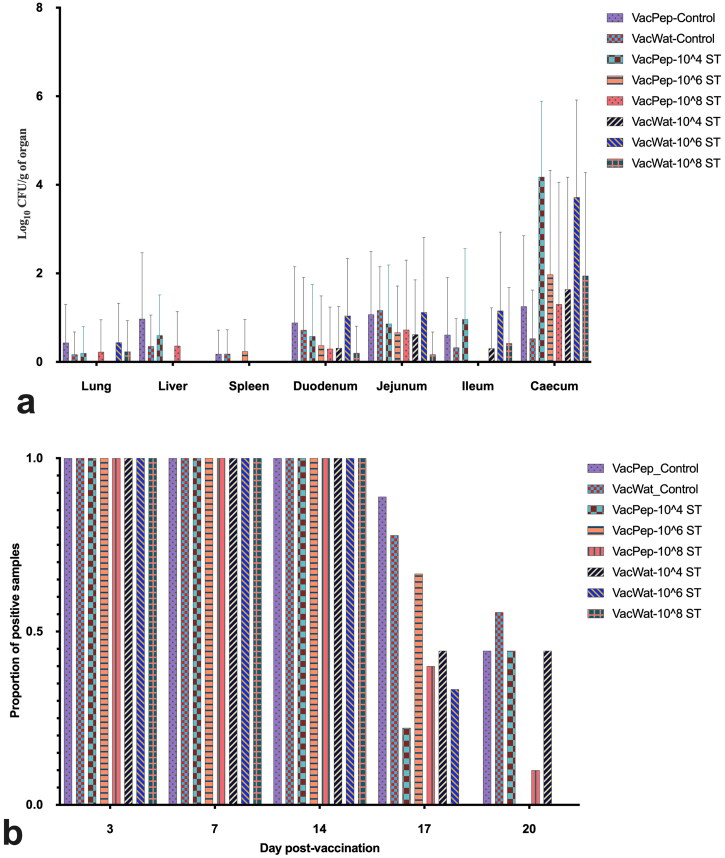
Vaccine detection in organs and cloacal swabs. a) Vaccine load in organs at day 20 post-vaccination. b) Vaccine proportion of positive samples at days 3, 7, 14, 17, and 20 post-vaccination. Broilers in the respective treatment groups were vaccinated at day 1 post-hatch and organs were collected for vaccine quantification at day 20, while cloacal swabs for vaccine detection at days 3, 7, 14, 17, and 20 post-vaccination. No vaccine was quantified from the unvaccinated control treatment groups; therefore, they were excluded from the graphic presentation of the data.

A very low load of vaccine (< 1 log_10_ CFU/g of organ) was detected at day 20 post-vaccination from lung, liver, spleen, jejunum and ileum using the direct plating method (Figure 2a). Comparison of different segments of the gut, showed that the caecum had higher loads of vaccine than other regions of the gut. Vaccine colonised 100% of the vaccinated chickens up to day 14 post-vaccination (Figure 2b). Vaccine detection in the gut through cloacal swabs significantly decreased after *S.* Typhimurium challenge. At day 20 post-vaccination, vaccine was detectable in the gut (through cloacal swabs) in 45% of the vaccinated control groups both in the peptone and water diluent treatment groups, whereas in the vaccinated and *Salmonella* infected groups, vaccine could be detected in the gut of approximately 45% of the chickens that were challenged with 10^4^ CFU of wild-type *S.* Typhimurium (Figure 2b). No vaccine was detectable in the faeces of the water or peptone diluent vaccinated chickens challenged with 10^6^ CFU of wild-type *S.* Typhimurium at day 20 post-vaccination. For the water diluent vaccinated chickens and challenged with 10^6^ or 10^8^ CFU of wild-type *S.* Typhimurium, no vaccine was detected in the faeces at day 20 post-vaccination (Figure 2b).

**Figure 3.**
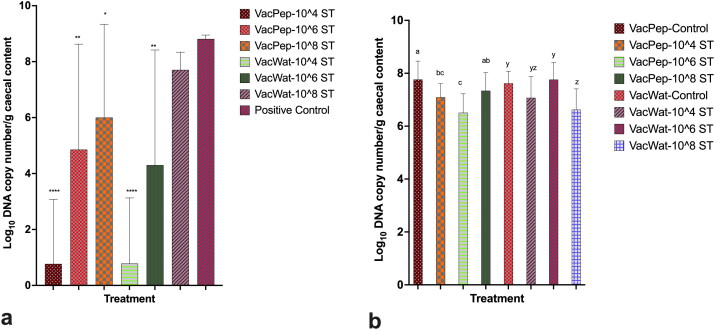
Vaccine and wild-type *S.* Typhimurium load in caecal contents of broilers quantified through qPCR at day 20 post-vaccination. a) Load of wild-type *Salmonella* in caecal contents at day 20 post-vaccination (day 7 post- wild-type *Salmonella* challenge). b) vaccine load in caecal content at day 20 post-vaccination. No vaccine was quantified from negative or positive control groups. No wild-type *Salmonella* was quantified from the negative or vaccine control groups. Therefore, where appropriate, unvaccinated or unchallenged groups were excluded from graphic presentation of the data, as no vaccine or wild-type *Salmonella* were quantified through qPCR from them. In the panel graphs, where appropriate, VacPep refers to the chicken treatment group that received vaccine reconstituted in peptone, while VacWat refers to the chicken treatment group that received vaccine reconstituted in water. ST refers to wild-type *S.* Typhimurium. In Figure 3a, within peptone diluent vaccinated and *Salmonella* challenged treatment groups (VacPep) asterisks (*, ** and ****) show significantly lower load of wild-type *S.* Typhimurium compared with the positive control group. In Figure 3a, within water diluent vaccinated and *Salmonella* challenged treatment groups (VacWat), asterisks (* and ****) show significantly lower load of wild-type *S.* Typhimurium compared with the positive control group. Asterisks (*, ** and ****) represent *P* values less than 0.0316, 0.005 and 0.00001, respectively. In Figure 3b, alphabets (a, b and c) show significantly lower level of vaccine recovery from treatment groups that received vaccine prepared in peptone diluent and challenged with different doses of wild-type *S.* Typhimurium compared with vaccinated peptone control (VacPep-control). In Figure 3b, alphabets (y and z) show significantly lower level of vaccine recovery from treatment groups that received vaccine prepared in water diluent and challenged with different doses of wild-type *S.* Typhimurium compared with vaccinated water control (VacWat-control).

The wild-type and vaccine *S.* Typhimurium load in the caecal content at day 20 post-vaccination was quantified through qPCR. For the peptone reconstituted vaccine, a significantly lower wild-type *S.* Typhimurium load was recorded for the 10^4^, 10^6^, and 10^8^ CFU challenge groups compared with the positive control (Figure 3a). For the water reconstituted vaccine, a significantly lower wild-type *S.* Typhimurium load was recorded for the 10^4^ and10^6^ treatment groups but not 10^8^ CFU treatment group compared with the positive control (Figure 3a). The vaccine load at day 20 post-vaccination was quantifiable from the caecal contents of all the vaccinated controls and vaccinated and wild-type *S.* Typhimurium challenged groups (Figure 3b).

**Figure 4.**
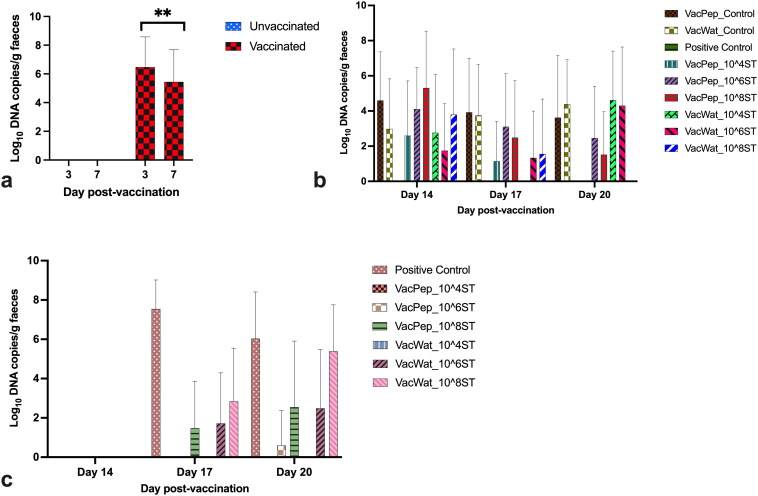
Vaccine and wild-type *Salmonella* load in faeces collected at different intervals from broilers. a) Vaccine load at days 3 and 7 post-vaccination prior of wild-type *Salmonella* challenge. b) Vaccine load at days 14, 17, and 20 post-vaccination (days 1, 4, and 7 post wild-type *Salmonella* Typhimurium challenge). c) wild-type *Salmonella* load at days 14, 17, and 20 post-vaccination (days 1, 4, and 7 post wild-type *S.* Typhimurium challenge). Both the vaccine and wild-type *Salmonella* load was quantified by qPCR. No vaccine was detected in negative or positive control groups. No wild-type *Salmonella* was detected in the negative or vaccine control groups. Therefore, where appropriate, unvaccinated or unchallenged groups were excluded from graphic presentation of the data, as no vaccine or wild-type *Salmonella* were quantified through qPCR from them. In the graph panels, where appropriate, VacPep refers to the chicken treatment group that received vaccine reconstituted in peptone, while VacWat refers to the chicken treatment group that received vaccine reconstituted in water. ST refers to wild-type *S.* Typhimurium.

The vaccine load quantified by qPCR from faecal samples collected at days 3, 7, 14, 17, and 20 post-vaccination showed that colonisation of the vaccine in the gut was significantly higher at day 3 compared with day 7 post-vaccination (Figure 4a). Wild-type challenge was introduced at day 13 post-vaccination. At days 14, 17, and 20 post-vaccination, vaccine was quantifiable in faecal samples of all the vaccinated treatment groups with an overall load less than log_10_ 5 (Figure 4b). After 24 hrs of challenge, wild-type *S.* Typhimurium was not detected by qPCR from any of the treatment groups except positive control (Figure 4c); however, using the enrichment method, *Salmonella* was detected in more than 50% of the vaccinated and challenged chickens and 100% from the positive control chickens. Wild-type *S.* Typhimurium load at days 17 and 20 post-vaccination was significantly lower in the peptone vaccinated treatment groups that received inoculums at 10^4^, 10^6^ and 10^8^ CFU/g of dust. For the water vaccinated treatment groups, wild-type *S.* Typhimurium load was significantly lower (log_10_ DNA copy number 2.48) in the treatment group that received inoculum at 10^6^ CFU compared with the treatment group that received inoculum at 10^8^ CFU (log_10_ DNA copy number 5.38) per gram of dust (Figure 4c). From both the peptone and water diluent groups challenged with 10^4^ CFU, no wild-type *S.* Typhimurium could be detected by qPCR (Figure 4c).

### Measurement of Salmonella Typhimurium specific antibodies in plasma

**Figure 5.**
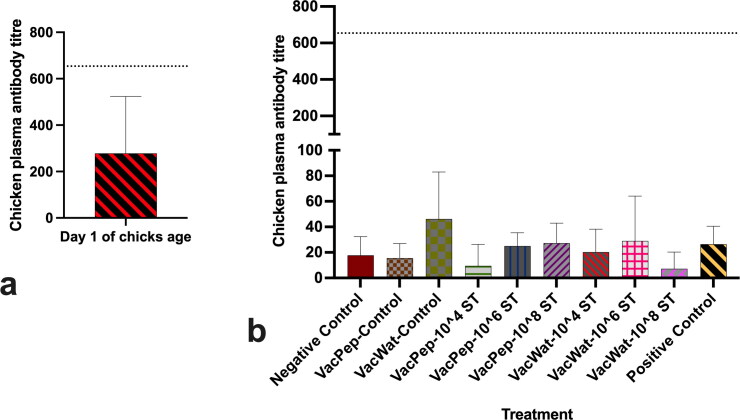
*Salmonella* Group B ELISA antibody titre level of broiler chickens. a) Antibody titre of plasma collected at day 1 of hatch prior to vaccination. b) Antibody titre of plasma samples collected at day 19 post-vaccination. In the x-axis legend of panel (b), VacPep refers to treatment groups that received vaccine reconstituted in peptone, while VacWat refers to treatment groups that received vaccine reconstituted in water. ST refers to wild-type *S.* Typhimurium challenged. Dotted lines at value 654 on Y-axis show titre value positive for *Salmonella* group B antibody.

The maternal antibody titres specific for *Salmonella* vaccine were below the positive threshold (Figure 5a). Furthermore, the data showed that neither the vaccine nor the wild-type *S.* Typhimurium infection resulted in any significant increases of antibody titre at day 19 post-vaccination (Figure 5b).

### Vaccine and wild-type S. **Typhimurium** had different effects on gut microbiota diversity

**Figure 6.**
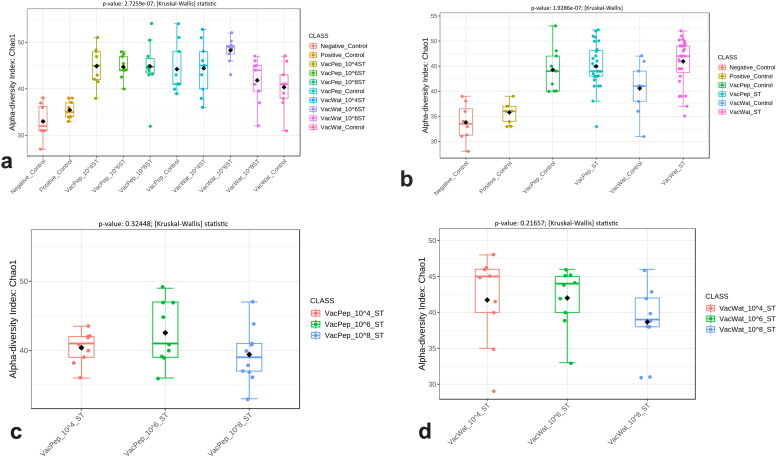
Alpha diversity of caecal microbiota of broiler chickens vaccinated and challenged with *S.* Typhimurium. a) Overall alpha diversity affected by vaccine diluent and different doses of wild-type *Salmonella* challenge. b) Pooled data showing effects of diluent and wild-type *Salmonella* challenge. c) *Salmonella* dose effect within peptone vaccinated groups. d) *Salmonella* dose effect within water vaccinated groups. Data for alpha diversity of the caecal microbiota were analysed in MicrobiomeAnalyst software by using non-parametric measurement. In Figure 6, VacPep refers to treatment groups that received vaccine reconstituted in peptone, while VacWat refers to treatment groups that received vaccine reconstituted in water. ST refers to wild-type *S.* Typhimurium.

Overall, there was a significant effect of vaccination and *S.* Typhimurium challenge on the alpha diversity of caecal microbiota. The vaccinated controls and vaccinated and wild-type *Salmonella* challenged groups showed significantly higher alpha diversity compared with the positive or negative control groups (Figure 6a). Data were also parsed by pooling together the different inoculum doses treatment groups (10^4^, 10^6^, and 10^8^ CFU) for each diluent. The alpha diversity was significantly higher in the vaccinated and *S.* Typhimurium challenged groups compared with the negative and positive controls (Figure 6b). Assessing the effects of dose of challenge, both in the peptone and water-based diluents, different challenge doses of *S.* Typhimurium did not significantly alter the alpha diversity of caecal microbiota (Figure 6c and d). In the absence of wild-type *S.* Typhimurium challenge, the vaccine diluent did not significantly affect (*p* = 0.19644) the alpha diversity (Supplementary Figure S2a). Compared with the negative control, there was no significant effect (*p* = 0.46552) of wild-type *S.* Typhimurium challenge on the alpha diversity of the gut microbiota of the unvaccinated and challenged chickens (Supplementary Figure S2b).

**Figure 7.**
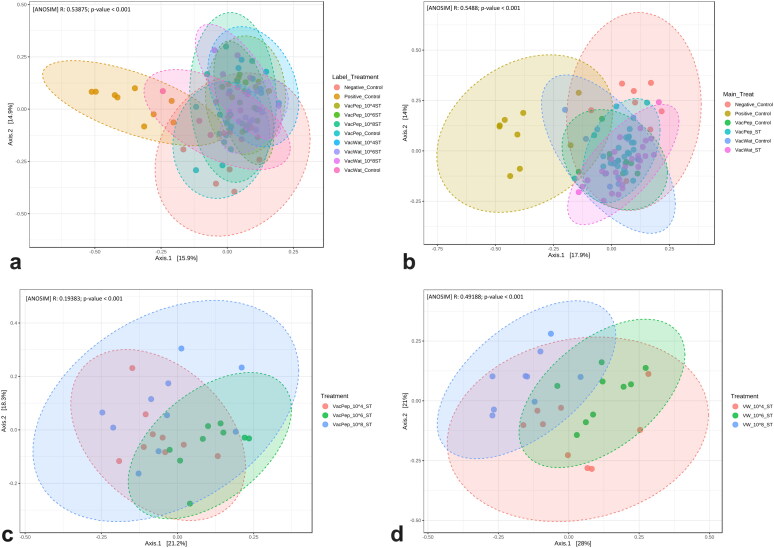
Beta diversity of caecal microbiota of broiler chickens vaccinated and challenged with *S.* Typhimurium. a) Beta diversity affected by vaccine diluent and wild-type *Salmonella* threshold of infection. b) Beta diversity affected by vaccine diluent and wild-type *Salmonella* challenge. c) Beta diversity affected by threshold of wild-type infection within peptone treatment group. d) Beta diversity affected by threshold of wild-type infection within water treatment group.

Beta diversity was significantly dissimilar between the vaccinated controls, vaccinated and wild-type *S.* Typhimurium challenged groups. Overall, both the vaccine diluent and different doses of *S.* Typhimurium significantly changed the beta diversity of caecal microbiota (Figure 7a and b). Beta diversity was significantly dissimilar between the vaccinated and challenged chickens that received different doses of wild-type *S.* Typhimurium irrespective of the diluent (Figure 7c and d). *S.* Typhimurium challenge significantly changed the beta diversity, as shown between the negative and positive control groups (Supplementary Figure S3a), whereas it was not different (*p* > 0.3750) between the chickens that received the vaccine reconstituted in water and peptone diluents (Supplementary Figure S3b).

### Vaccinated and challenged chickens showed different microbial abundance

**Figure 8.**
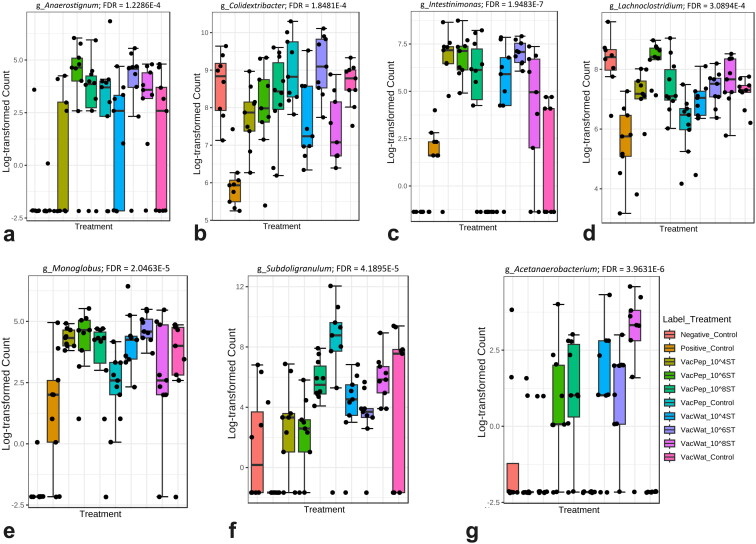
Abundance of genera in the caecal content of broilers in response to vaccine and wild-type *S.* Typhimurium threshold of infection. a) *Anaerostignum*, b) *Colidextribacter*, c) *Intestinimonas*, d) *Lachnoclostridium*, e) *Monoglobus,* f) *Subdoligranulum,* and g) *Acetanaerobacterium*. The abundance was measured at genus level in MicrobiomeAnalyst using a single factor analysis option with FDR < 0.05 for determining the level of significance.

Overall, a total of 47 microbial genera differed in abundance in association with vaccine administration and wild-type *S.* Typhimurium challenge. In general, the microbiota profile of the vaccinated birds was different from birds challenged with wild-type *S.* Typhimurium. Overall, the vaccinated controls and vaccinated and wild-type challenged chickens showed significantly higher abundance of *Acetanaerobacte­rium*, *Anaerostignum*, *Colidextribacter*, *Intestinimonas*, *Lachnoclostridium*, *Monoglobus* and *Subdoligranulum* (Figure 8a–g).

Within each diluent, the threshold of infection significantly affected the relative abundance of < 4 individual genera. For example, in the water diluent group, the abundance of *Dickeya* and *Oscillibacter* decreased while *Sellimonas* increased in the chickens that received the higher infection dose (10^8^ CFU) of wild-type *Salmonella* (Supplementary Figure S4a–c). In the peptone diluent group, the abundance of the only significant genus, *Fournierella* was significantly lower in the 10^8^ infected group compared with the 10^4^ and 10^6^ CFU treatment groups (Supplementary Figure S4d). Both vaccine and wild-type *S.* Typhimurium reduced the abundance of *Flavonifractor*. Assessing the effects of wild-type *S.* Typhimurium challenge on gut microbiota, the abundance level of *Lachnoclostridium*, *Flavonifractor*, *Colidextribacter* and *Clostridium*_*innocuum*_group was significantly (FDR < 0.05) lower in the challenged compared with the negative control group (Supplementary Figure S5a–d).

## Discussion

In Australia, Vaxsafe® ST is a registered vaccine for control of foodborne *S*. Typhimurium in poultry. The vaccine strain was produced by the disruption of the *aroA* gene, which renders the vaccine candidate unable to produce the aromatic amino acids required for the normal growth of the bacterium. Based on the *aroA* disruption mechanism, we hypothesised that Vaxsafe® ST may be primed for colonisation in the chicken gut if reconstituted in peptone. The rationale for pre-warming the diluent to 41 °C was to mimic the body temperature of chickens. Our *in vitro* pilot study (unpublished data) showed that peptone enhanced the invasion of Vaxsafe® ST strain into Caco2 epithelial cells and it was hypothesised that this may translate into improved *in vivo* performance. Therefore, the main objective of this study was to understand if a nutrient rich diluent (i.e. peptone) would effectively prime the vaccine for better gut colonisation and invasion into internal organs than if reconstituted in a nutrient poor diluent (i.e. water). It was also hypothesised that the vaccine would be more effective when chickens were challenged with lower inoculum doses of wild-type *S.* Typhimurium.

Vaccine was recovered from all vaccinated chickens until day 14 post-vaccination demonstrating that Vaxsafe® ST effectively colonised and persisted in the gut of the broilers. Following wild-type *S.* Typhimurium challenge at day 13 post-vaccination, the shedding level of vaccine reduced but could still be detected and or quantified mainly from the vaccine controls and vaccinated and 10^4^ CFU wild-type *Salmonella* challenged treatment groups until the termination of the experiment (day 20 post-vaccination). The higher rate of recovery of the vaccine strain from the treatment groups that received a lower *S.* Typhimurium challenge (10^4^ CFU/pen) compared with the 10^8^ CFU/pen treatment groups showed the better protection of chickens exposed to low loads of wild-type *S.* Typhimurium. The vaccine load in organs at day 20 post-vaccination data showed that the colonisation rate of Vaxsafe® ST was significantly higher in the caeca compared with duodenum, jejunum, ileum, liver and spleen. This observation is consistent with previous studies (Bohez et al. 2007; Jia et al. 2023). Using peptone as a diluent for vaccine reconstitution showed positive effects on protection of chickens against wild-type *S.* Typhimurium challenge. For example, a numerically lower load of wild-type *S.* Typhimurium was recorded in the lungs of chickens that received vaccine reconstituted in peptone. Among the internal organs, lung had a lower load of wild-type *S*. Typhimurium, this could be due to pulmonary cellular defences in avian lung but further research is required to confirm this hypothesis. Additionally, both the qPCR and direct plating culture methods showed a significantly lower load of wild-type *S.* Typhimurium in the caeca of chickens that received vaccine reconstituted in peptone. However, the shedding level of the vaccine in the faeces at days 3, 7, and 14 post-vaccinations remained the same for the peptone and water diluents vaccinated treatment groups.

In the current study, a contaminated dust method was used to introduce a wild-type *S.* Typhimurium challenge to the selected treatment groups using 10^4^, 10^6^, and 10^8^ CFU/g of dust sprinkled in a pen. The use of enriched faecal samples for detection of wild-type *S.* Typhimurium showed that the model worked effectively, and the bacteria were detected after day 1 (24 hr) post-challenge. Studies have previously reported that *Salmonella* Heidelberg and *Salmonella* Enteritidis can be recovered from chickens after 1 day post-challenge (Van Immerseel et al. 2002; Knap et al. 2011). In the current study, the qPCR data of wild-type *S.* Typhimurium quantification after day 1 post-challenge showed that the bacterial load in cloacal swabs was below the limit of detection; therefore, could not be quantified. However, at day 4 post-challenge, the load of *Salmonella* could be quantified with a higher count from the unvaccinated and challenged (positive control) group.

The overall reduction of wild-type *S.* Typhimurium in vaccinated chickens suggests vaccine mediated protection. In liver, spleen, duodenum, jejunum, ileum and caecum, the load of wild-type *S.* Typhimurium was significantly lower in all the vaccinated and challenged chickens using 10^4^ and 10^6^ CFU/pen inoculum dose. This indicated that irrespective of the diluents used, vaccination tended to significantly reduce the load of wild-type *S.* Typhimurium in the different gut segments, spleen, and liver. The faecal data at days 14, 17, and 20 post-vaccination showed that collectively the shedding percentage for wild-type *S.* Typhimurium from the 10^4^, 10^6^ and 10^8^ CFU vaccinated treatment groups was 26, 50, and 89, respectively. In *Salmonella* challenge studies, it has been shown that even a low inoculum dose of *Salmonella* in the gut replicates exponentially (Sadeyen et al. 2004). The wild-type *S.* Typhimurium load quantified through qPCR from the caecal contents at day 20 post-vaccination also showed that at 10^4^ and 10^6^ CFU doses of infection, vaccine better protected the chickens as evident by significantly lower load of *S.* Typhimurium in the 10^4^ and 10^6^ CFU/pen treatment groups. Overall, the data obtained from the current study support the notion that Vaxsafe® ST better protected broilers from wild-type *S.* Typhimurium at lower doses of infection.

In the present study, fertile eggs incubated for commercial broiler hatching were sourced from a hatchery that vaccinate breeder flocks for *Salmonella* Typhimurium. The negative status of day-old chicks for *Salmonella* specific maternal antibodies in the blood showed that there was no likelihood of antibodies neutralisation in the day-old chicks vaccinated with Vaxsafe® ST. In a previous study, an intramuscular injection of Vaxsafe® ST in 6 weeks old broilers resulted in significantly higher antibody titres at week 2 post-vaccination (Groves et al. 2015).

The gastrointestinal system carries microbial communities that play a fundamental role in host digestion, competitive exclusion of pathogens, and regulation of the mucosal immune system. Chicken caeca not only act as a site for fermentation but also harbours many different microbes with other functional characteristics (Sergeant et al. 2014). Two types of *S.* Typhimurium vaccines in broilers have been shown to considerably change the caecal microbiota structure (Park et al. 2017). In this study, the alpha diversity index showed that Vaxsafe® ST administration resulted in different diversity than seen in unvaccinated animals, which remained consistent post wild-type *S.* Typhimurium challenge compared with the unvaccinated (negative control) and *Salmonella* challenge (positive control) treatment groups. Within each vaccine diluent (peptone and water) treatment groups, the non-significant difference in Chao 1 alpha diversity index between the different infection doses (10^4^, 10^6^, and 10^8^ CFU/pen) of wild-type *Salmonella* showed that the alpha diversity of the gut of the vaccinated chickens was not dependent on dose of *S.* Typhimurium. Peptone and water diluents minimally affected the gut microbiota structure of individual chickens. Beta diversity index compared the community structure between individuals. A significant dissimilarity in the index of beta diversity between the unvaccinated, vaccinated only, challenged only and vaccinated and challenged treatment groups showed that both the vaccine and *S.* Typhimurium challenge influenced the structure of gut microbiota. Although the treatment groups overlapped, the positive control group clustered separately, indicating that wild-type *S.* Typhimurium changed the gut microbiota in a way that was different from both the unvaccinated and unchallenged and vaccinated and challenged groups. Within each diluent, different threshold of infection doses of wild-type *S.* Typhimurium changed the beta diversity differently. Both peptone and water diluents did not alter the beta diversity of the microbial population.

Changes in beta diversity are reflective of changes in abundance of individual taxa. Overall, the abundance of some genera was affected by the wild-type *S.* Typhimurium challenge in the absence of vaccination. In the vaccinated chickens, wild-type *S.* Typhimurium challenge minimally affected the abundance compared with the unvaccinated and challenged group (positive control). Within the water diluent treatment groups, a higher inoculum dose of wild-type *Salmonella* reduced the relative abundance of *Dickeya* and *Oscillibacter*, and increased *Sellimonas* population. Within the peptone diluent treatment groups, *Fournierella* was the only genus that reduced in relative abundance in the higher inoculum dose of wild-type *S.* Typhimurium. This could indicate that although beta diversity was minimally affected, peptone as a diluent is effective. The *Dickeya* population in the poultry environment and excreta has been associated with high performance of broilers (Bindari et al. 2021). *In silico* analysis has shown the effectiveness of *Dickeya* as a pectinase producer in broiler poultry digestion (Dittoe et al. 2020).

## Conclusions

The current study has demonstrated that Vaxsafe® ST effectively colonised the gut of broiler chickens and resulted in reduced shedding levels of wild-type *S.* Typhimurium in a dust infection model. The two vaccine diluents (peptone and water) showed comparable effects on protection of chickens against wild-type *S*. Typhimurium. The pattern of changes in gut microbiota due to vaccination was different from the changes observed post wild-type *S.* Typhimurium infection.

## Supplementary Material

Supplementary data.docx

## Data Availability

The 16S rRNA gene sequence data are available from the Sequence Read Archive (SRA) database under the accession number PRJNA1058241.
