# Supplementary material for: Vaccine protection of broilers against various doses of wild-type Salmonella Typhimurium and changes in gut microbiota
Source: Vet Q. 2024 Dec 25;45(1):1–14. doi: 10.1080/01652176.2024.2440428 (PMC11703527; doi:10.1080/01652176.2024.2440428)
Supplement: Supplementary data.docx [file TVEQ_A_2440428_SM1765.docx]

**Vaccine protection of broilers against various doses of wild-type *Salmonella* Typhimurium and changes in gut microbiota**

**Samiullah Khan^a^, Andrea R. McWhorter^a^, Nicky-Lee Willson^a^, Daniel M. Andrews^b^, Gregory J. Underwood^b^, Robert J. Moore^c^, Thi Thu Hao Van^c^, and Kapil K. Chousalkar^a^***

^a^School of Animal and Veterinary Sciences, The University of Adelaide, Roseworthy, South Australia, 5371, Australia

^b^Bioproperties Pty Ltd, Ringwood, Victoria, 3134, Australia

^c^RMIT University, School of Science, Bundoora, Victoria, 3083, Australia

**Supplementary data**

**
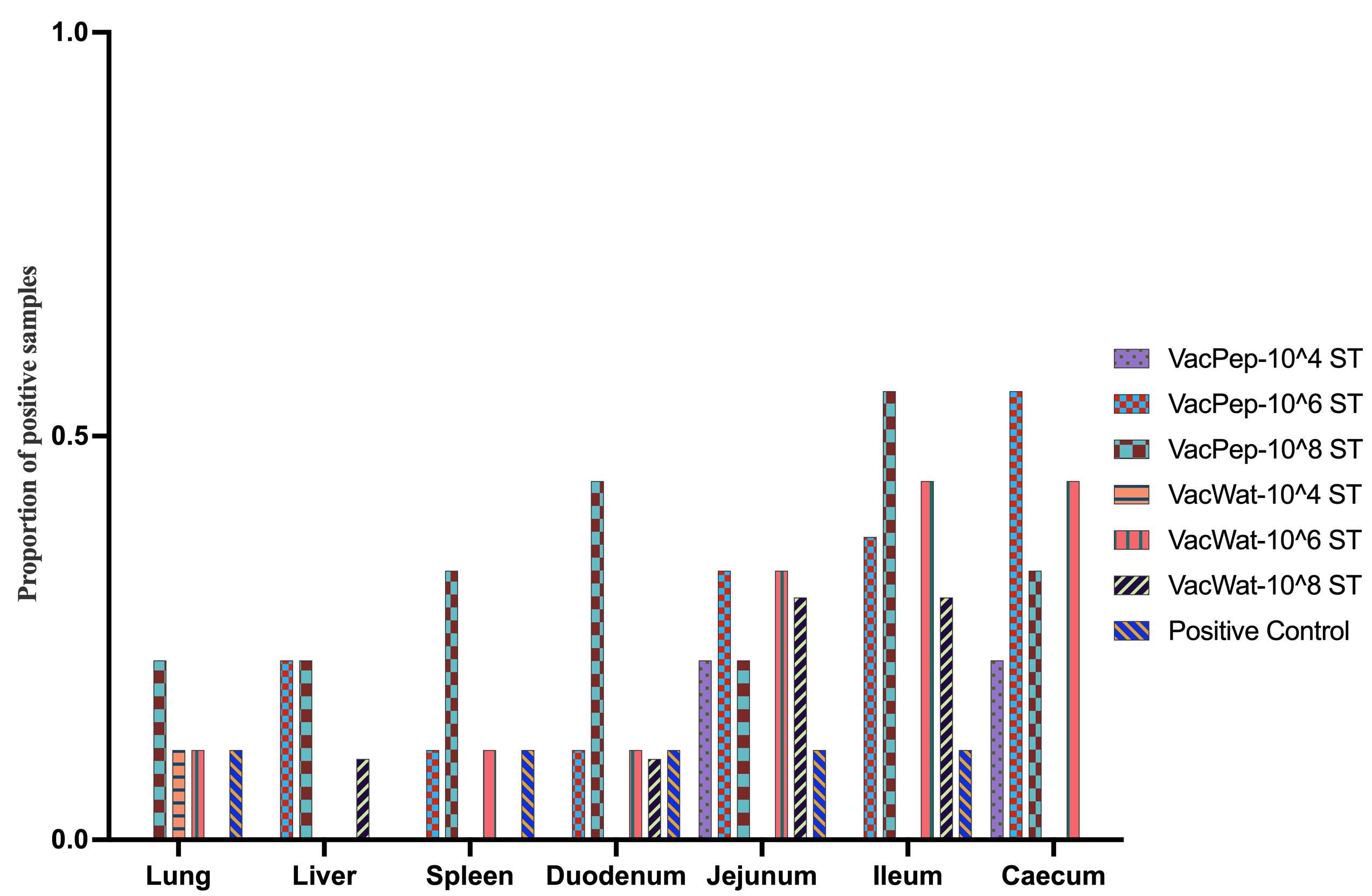
**

**Supplementary Figure S1. Recovery percentage of wild-type *Salmonella* from organs that did not show any growth on agar upon direct plating.** Only organs that did not show colonies on XLD agar plates were enriched in BPW and RVS and assessed for *Salmonella* recovery using “0” as no growth and “1” as confirmed growth.


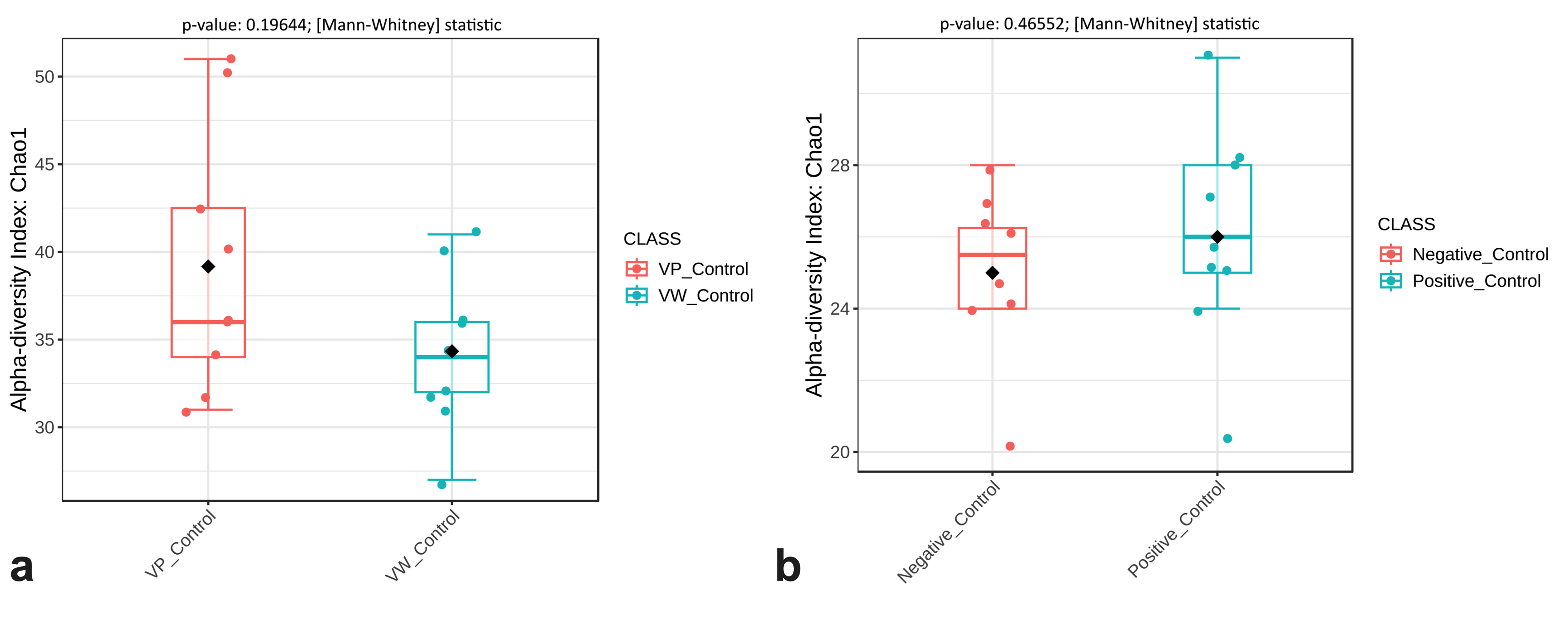


**Supplementary Figure S2. Effect of vaccine diluent and wild-type *Salmonella* challenge on alpha diversity of caecal microbiota of broilers. a)** Comparing the effects of peptone and water as diluents for vaccine reconstitutions and their effects on beta diversity. **b)** Caecal content alpha diversity of the negative control and wild-type *Salmonella* infected chickens.

**
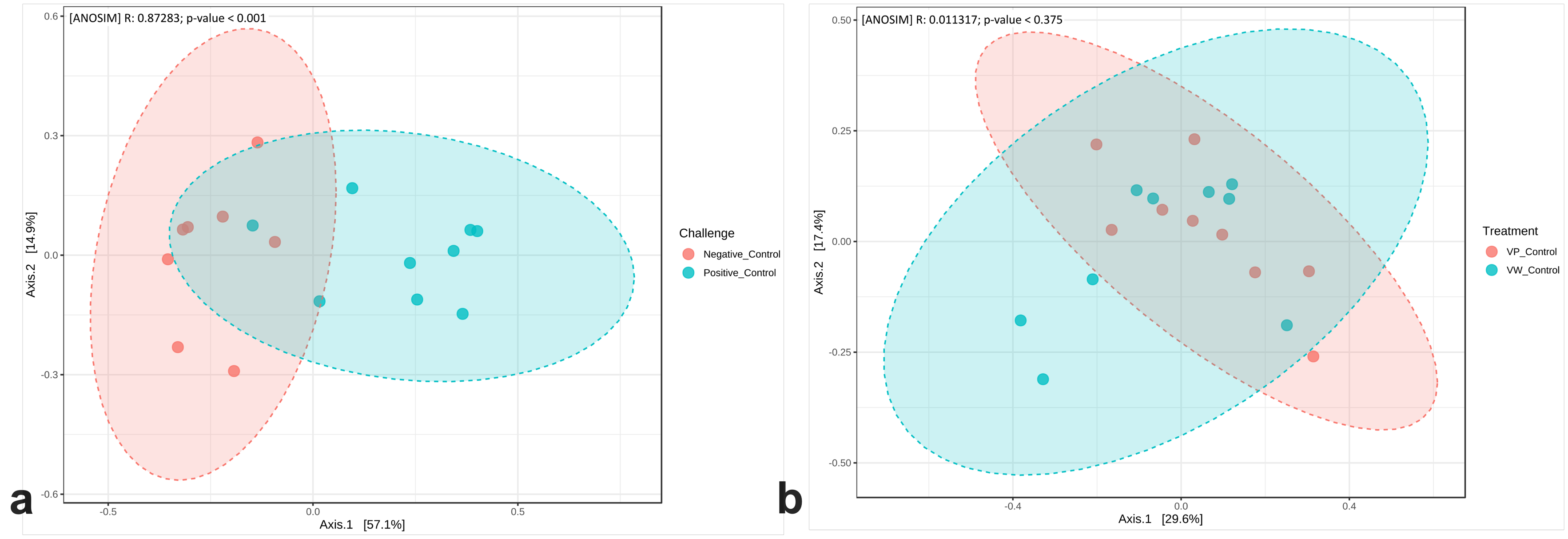
**

**Supplementary Figure S3: Beta diversity of caecal microbiota affected by *S.* Typhimurium challenge and vaccination. a)** Beta diversity of control and *Salmonella* challenged chickens. **b)** Beta diversity of vaccinated chickens where vaccine was reconstituted in peptone and water diluents prior to administration.

**
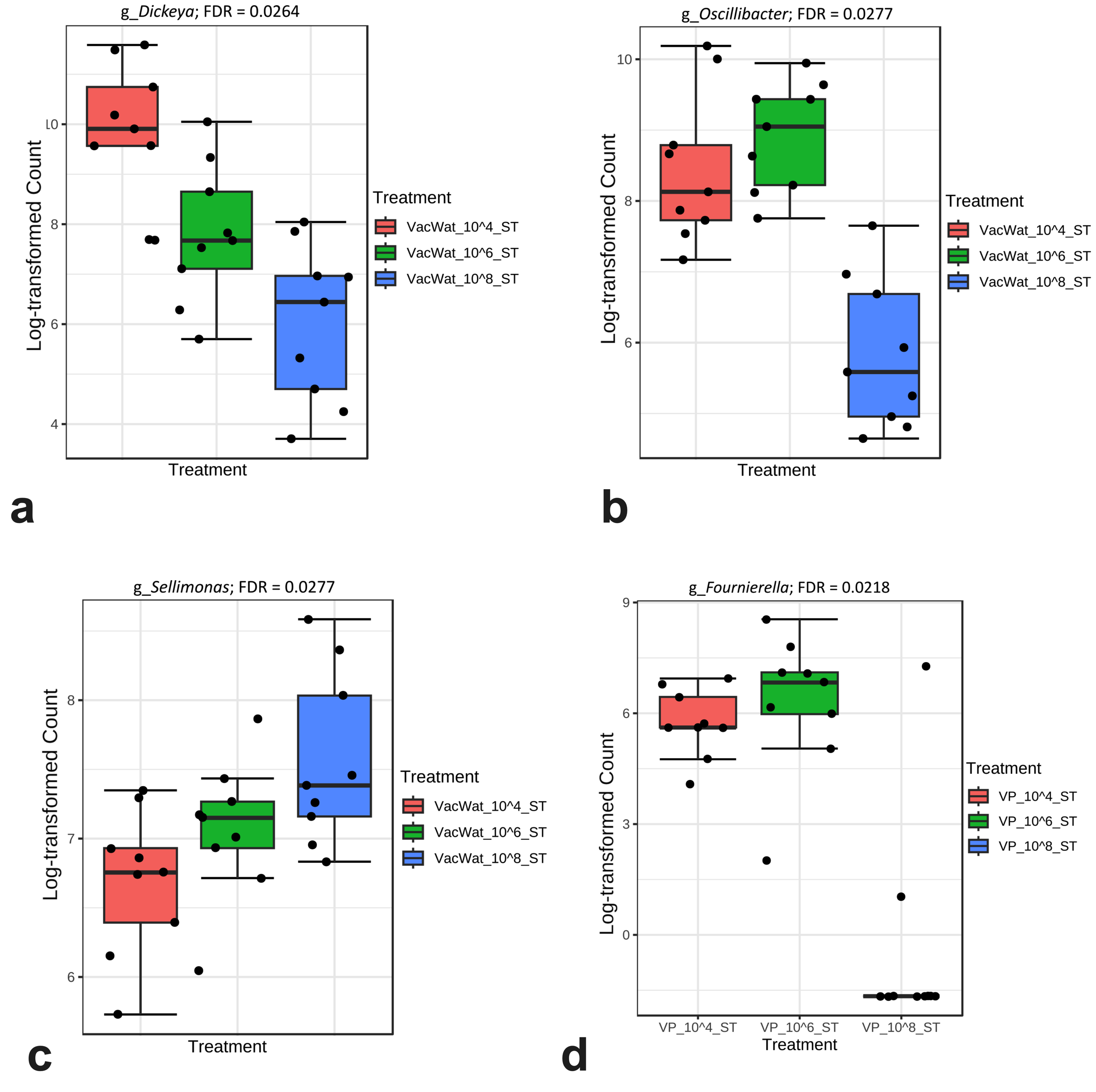
**

**Supplementary Figure S4. Variation in microbial abundance of caecal content affected by threshold of wild-type *Salmonella* infection within each diluent.** Abundance levels of **a)** *Dickeya*, **b)** *Oscillibacter* and **c)** *Sellimonas* affected by threshold of infection of wild-type *Salmonella* in water based diluent vaccinated chickens. **d)** Abundance level of *Fournierella* affected by threshold of infection of wild-type *Salmonella* in peptone-based diluent vaccinated chickens.

**
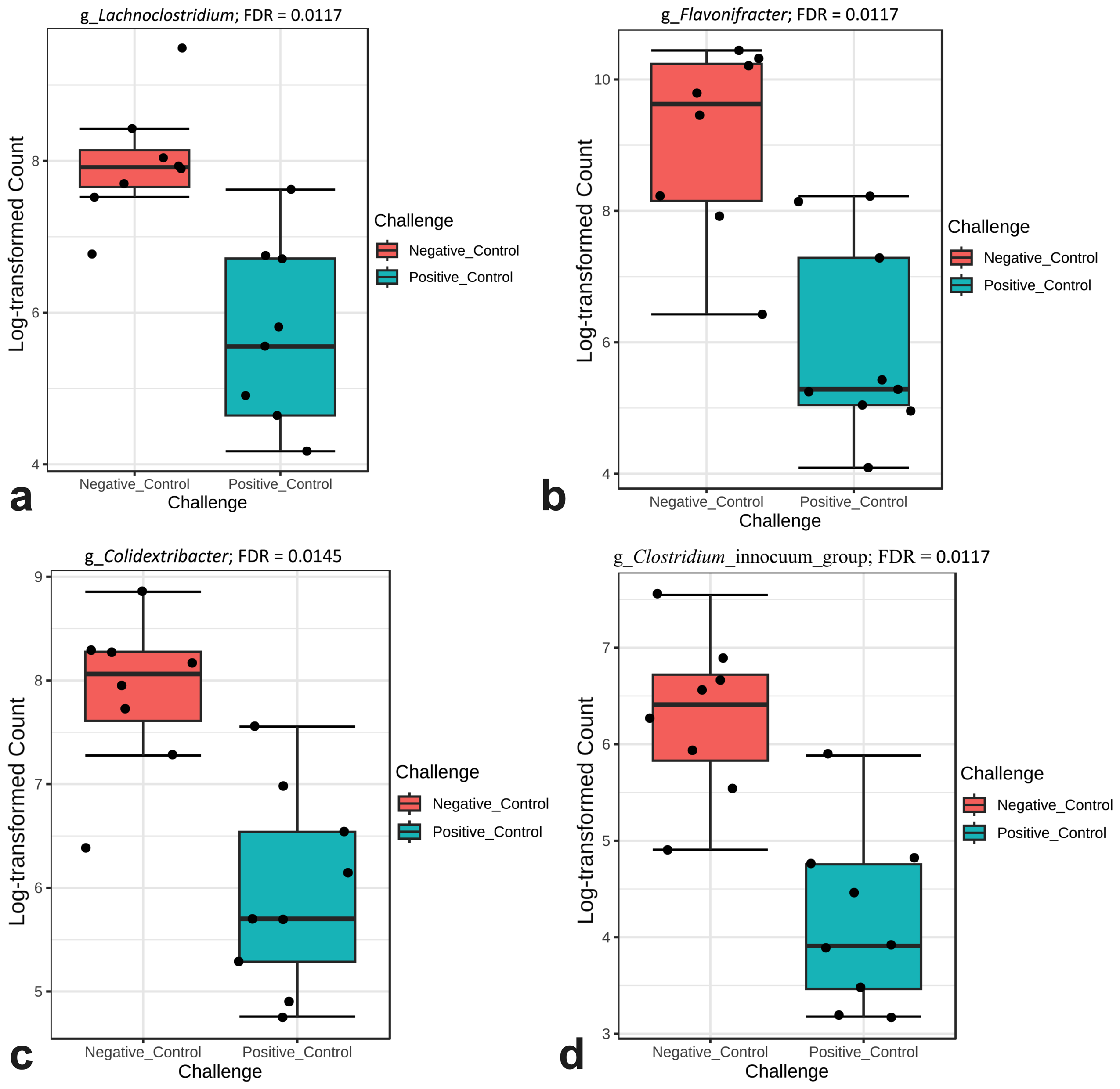
**

**Supplementary Figure S5. Variation in microbial abundance of caecal content between *S.* Typhimurium challenged and negative control broilers. a)** *Lachnoclostridium*, **b)** *Flavonifractor*, **c)** *Colidextribacter* and **d)** *Clostridium*_innocuum_group.
